# Effect of on-demand vs continuous prescription of proton pump inhibitors on symptom burden and quality of life: results of a real-world randomized controlled trial in primary care patients with gastroesophageal reflux disease

**DOI:** 10.1080/07853890.2024.2354683

**Published:** 2024-05-16

**Authors:** Anna Andreasson, Lars Agréus, Nikolaos Mastellos, Grzegorz Bliźniuk, Dorota Waśko-Czopnik, Agapi Angelaki, Eirini Theodosaki, Christos Lionis, Karin Hek, Robert Verheij, Ellen Wright, Stevo Durbaba, Jean Muris, Piotr Bródka, Stanislaw Saganowski, Jean-Francois Ethiér, Vasa Curcin, Brendan Delaney

**Affiliations:** a Division of Psychobiology and Epidemiology, Department of Psychology, Stockholm University, Stockholm, Sweden; b Unit of Clinical Medicine, Department of Medicine Solna, Karolinska Institutet, Stockholm, Sweden; c School of Psychological Sciences, Macquarie University, NSW, Australia; d Division for Family Medicine and Primary Care, Department of Neurobiology, Care Sciences and Society, Karolinska Institutet, Huddinge, Sweden; e Department of Primary Care and Public Health, School of Public Health, Imperial College London, London, UK; f Faculty of Cybernetics, Military University of Technology, Warsaw, Poland; g Department of Gastroenterology and Hepatology Medical University Wroclaw ul. Borowska 213, Wroclaw; h Clinic of Social and Family Medicine, School of Medicine, University of Crete, Greece, Heraklion; i Nivel, Netherlands Institute for Health Services Research, Utrecht, the Netherlands; j Tilburg School of Social and Behavioral Sciences, Tilburg University, Tilburg, the Netherlands; k Health Care Institute Netherlands, Diemen, the Netherlands; l School of Life Course & Population Sciences, Faculty of Life Sciences and Medicine, King’s College London, London, UK; m Department of Family Medicine, Maastricht University, Care and Public Health Research Institute, Maastricht, The Netherlands; n Department of Artificial Intelligence, Wroclaw University of Science and Technology, Wroclaw, Poland; o Groupe de recherche interdisciplinaire en informatique de la santé (GRIIS.ca), Université de Sherbrooke, Sherbrooke, Canada; p Department of Informatics, King’s College London, London, UK; q Department of Department of Surgery and Cancer, Imperial College London, London, UK

**Keywords:** gastroesophageal reflux disease, proton pump inhibitors, continuous use, on-demand use, randomized controlled trial, quality of life, self-rated health

## Abstract

**Objectives:**

This study aimed to assess the impact of on-demand versus continuous prescribing of proton pump inhibitors (PPIs) on symptom burden and health-related quality of life in patients with gastroesophageal reflux disease (GERD) presenting to primary care.

**Methods:**

Thirty-six primary care centres across Europe enrolled adult GERD patients from electronic health records. Participants were randomised to on-demand or continuous PPI prescriptions and were followed for 8 weeks. PPI intake, symptom burden, and quality of life were compared between the two groups using mixed-effect regression analyses. Spearman’s correlation was used to assess the association between changes in PPI dose and patient-reported outcomes.

**Results:**

A total of 488 patients (median age 51 years, 58% women) completed the initial visit, with 360 attending the follow-up visit. There was no significant difference in PPI use between the continuous and on-demand prescription groups (*b*=.57, 95%CI:0.40-1.53), although PPI use increased in both groups (*b* = 1.33, 95%CI:0.65 − 2.01). Advice on prescribing strategy did not significantly affect patient-reported outcomes. Both symptom burden (Reflux Disease Questionnaire, b=-0.61, 95%CI:-0.73 − -0.49) and quality of life (12-item Short Form Survey physical score *b* = 3.31, 95%CI:2.17 − 4.45) improved from baseline to follow-up in both groups. Increased PPI intake correlated with reduced reflux symptoms (*n* = 347, ρ=-0.12, *p* = 0.02) and improved quality of life (*n* = 217, ρ = 0.16, *p* = 0.02).

**Conclusion:**

In real-world settings, both continuous and on-demand PPI prescriptions resulted in similar increases in PPI consumption with no difference in treatment effects. Achieving an adequate PPI dose to alleviate reflux symptom burden improves quality of life in GERD patients. EudraCT number 2014-001314-25.

## KEY MESSAGES

- Continuous and on-demand prescription increase in proton pump inhibitor consumption equally in real-world settings and did not result in different outcomes.
- Reaching a sufficient dose of proton pump inhibitor to reduce reflux symptom burden improves quality of life in patients with gastroesophageal reflux disease.

## Introduction

Gastroesophageal reflux disease (GERD) encompasses disorders primarily resulting from the backward flow of stomach acid into the oesophagus, leading to symptoms and/or damage to the oesophageal lining. The most common symptoms include heartburn and acid regurgitation, (characterized by a bitter burning sensation at the back of the mouth) [1]. The spectrum of GERD [2] ranges from non-erosive reflux disease (which lacks visible oesophageal erosions) [3], erosive esophagitis (that can occur without symptoms) [4] of the squamous mucosa, Barrett’s esophagitis [5], to adenocarcinoma of the distal oesophagus [6,7].

As reflux symptoms are common, and for some people harmless, GERD is defined as a disease when quality of life is affected [2]. Prevalence in Europe and North America is reported between 10% and 20% [8]. The notion of an increase in GERD prevalence is supported by a longitudinal study from Sweden, showing an absolute increase in GERD by 2% per decade since 1988 [9]. Factors contributing to this rise include increasing obesity rates (higher intra-abdominal pressure), decreasing prevalence of *Helicobacter pylori* in the stomach, and associated decrease in gastric corpus atrophy, which reduces acid secretion [10].

GERD is primarily treated in primary care or over-the-counter by pharmacists. Standard pharmacological treatment of GERD is the lowest effective dose of PPI per day (i.e. continuous use) or ‘on demand’ (i.e. when the patient experiences bothersome symptoms of GERD). Research presents conflicting views on the effectiveness of these approaches [11], suggesting that an on-demand regimen might reduce costs and minimize long-term side effects [12]. However, this could result in increased symptom perception, poorer quality of life, and greater disease burden for some patients.

Previous research has primarily focused on treatment adherence and symptom management, comparing willingness to continue treatment and symptom reports between continuous versus on-demand prescription [13]. Few studies have explored the impact of treatment regimens on quality of life, and none have compared self-rated health between continuous and on-demand PPI. Self-rated health is a highly relevant outcome as poor self-rated health is an independent predictor of morbidity, health care consumption, sick leave, and mortality [14–16].

This study aims to assess how prescription patterns (continuous vs. on-demand) affect symptom severity, health-related quality of life, and self-rated health in primary care patients using PPIs. Specifically, it seeks to determine if on-demand PPI use results in greater symptom burden and poorer health-related quality of life compared to continuous use. Additionally, the study will evaluate a new real-world trial platform, the TRANSFoRm project, which integrates clinical trial processes into routine care through electronic health records [17].

## Material and methods

The study is a registered clinical trial EudraCT nr 2014-001314-25 and was conducted between May and December 2015. Ethical approval was sought and granted in all participating countries: Greece – the Ethics Committee of the General University Hospital PA.GNI. and “Venizelio” General Hospital (Protocol Number 9136/8-8-15, Decision Number 469), Poland – the Bioethics Committee in Wroclaw (Komisja Bioetyczna przy DIL we Wrocławiu, approval number 2/NT/2014, date of approval: 11.06.2014), the Netherlands – the medical ethics review committee of VU University Medical Center (approval number NL49118.029.14) and the United Kingdom (UK) – approval was given by the Health Research Authority (IRAS 156168, CPMS ID 18049). Written informed consent was given by all participants. All authors had access to the study data and reviewed and approved the final manuscript.

### Participants

Participants in this study were sourced from 36 primary care centres in four countries: 8 in Greece, 10 in Poland, 8 in the Netherlands, and 10 in the UK. Eligible patients were adults aged 18 to 65 years, who had been diagnosed with GERD after experiencing symptoms at least twice a week that adversely affected their quality of life, and who had previously found symptom relief from PPIs (PPI treatment responders). In other words, all participants were either current (taking PPIs at the time of the visit) or former PPI users. Patients with Barrett’s disease, severe esophagitis (Los Angeles grade C or D), a recent myocardial infarction, or who were pregnant were excluded due to potential impacts on GERD treatment outcomes and PPI efficacy.

In the Netherlands, potential participants were contacted *via* mail and invited to participate. In Greece, Poland and the UK recruitment was targeted at patients with GERD presenting for clinical consultations, regardless of the reason for the visit, to assess the impact of utilizing the TRANSFoRm platform on recruitment rates [17,18].

### Procedure

In the practices using the TRANSFoRm Study System [19–21], the general practitioner (GP) received a notification through the electronic health record system for patients meeting the inclusion criteria. In the other practices, the GP identified participants through manual selection. Upon obtaining consent and providing study information, GPs completed a Clinical Reported Outcome Measures (CROM) form during the initial visit. Patients were then randomized to either on-demand or continuous prescription of PPI using a built-in randomization algorithm within the TRANSFoRm platform and the reference electronic case report form (eCRF) used by reference primary care centres. Patients were randomized in four blocks based on sex and age (50 years and below, and above 50 years) [19] to either continuous prescription of PPI (20 mg Omeprazole daily) or on demand (20 mg Omeprazole on demand, maximum daily intake two pills, i.e. 40 mg). Patients filled out Patient Reported Outcome Measures (PROM) forms following their initial visit and again at an 8-week follow-up visit, during which future treatment plans were discussed with their GP. The tools and forms used in this study have been certified according to good clinical practice [17,18,22].

### TRANSFoRm study system

The TRANSFoRm Study System (TSS) aims to streamline controlled studies. The TSS allows the participation of many general practitioners (GPs) from different countries using various local electronic health record (EHR) systems. The users of the system are patients, GPs, and researchers. Patients interact with the TSS through mobile and web applications when they report their health. In the case of alarm symptoms, GPs will receive a notification in their local EHR system. Researchers can track the study’s progress on an ongoing basis and analyse the results through a web application [20].

Communication throughout the system is based on the Clinical Data Interchange Standards Consortium (CDISC) Operational Data Model (ODM) standard. The ODM model was extended to facilitate user interface consistency across mobile and web devices. The connection, authorization, and security within the TSS are ensured by many modules embedded as a middleware Enterprise Service Bus. The communication with EHR systems, on the other hand, is performed through Data Node Connectors - the mediator modules created for each EHR separately. The TSS was tested in several steps. At first, each module completed extensive unit testing. Next, the TSS was deployed to the test environment. After successfully passing all test scenarios, the system was deployed to the target environment. The test environment was maintained to allow for system development and testing of potential updates [17,21].

### Reference system

The reference system’s electronic case report forms (eCRFs) were developed using the Microsoft ASP.NET framework and validated according to a computer software validation methodology. The eCRFs were translated into Dutch, English, Greek, and Polish, with validation by native speakers from the study team, ensuring accuracy and comprehensibility across languages.

### Variables

#### Clinical reported Outcome Measures

CROMs were reported either through the TRANSFoRm Study System or through the reference eCRF at the time of the visit [22] and included current PPI dose (total number of PPI doses taken during the last week as reported by the patient), and body mass index (BMI, kg/m^2^). If the patient had had an upper endoscopy prior to study enrolment this was registered, and esophagitis status based on the Los Angeles criteria [2] was recorded.

#### Patient reported Outcome Measures

The patients completed the forms including questions on demographics, drug use, symptom burden, quality of life and self-rated health either using the TRANSFoRm smartphone or web application for PROMs [19,20] or on paper. The paper forms were filled out at the practice at the time of the visit. The forms filled out in the application could be filled out anywhere with internet access. If the patient using the TRANSFoRm platform forgot to fill the PROM form, the system sent reminders.

Symptom burden was assessed using the Reflux Disease Questionnaire (RDQ) that asks for frequency (absent to daily) and severity (absent to severe) of six reflux symptoms: 1) burning feeling behind breastbone, 2) pain behind breastbone, 3) burning feeling in centre of upper stomach, 4) pain in centre of stomach, 5) acid taste in mouth, and 6) unpleasant movement of material upwards from the stomach [23]. Health related quality of life was assessed using the Short Form 12 item (version 2) Health Survey (SF-12v2). The aggregated subscales of physical and mental health were calculated and used in the analyses [24]. Self-rated health was assessed using the following validated question: “How would you rate your general state of health?” and answered on a 5-point Likert scale as: Very good, good, neither good nor poor, poor or very poor [25]. Both questionnaires have been translated and validated in the local languages of the participating countries.

#### Statistics

Firstly, the effect of treatment allocation on PPI use was tested in an intention to treat analysis using a mixed effect regression model with treatment allocation (continuous versus on demand), visit (follow-up versus baseline), and an interaction term between treatment allocation and visit (difference between treatment allocation groups at follow-up) as exposure and PPI consumption as outcome, using identity as a random effect. In a follow-up analysis, PPI status at baseline and interaction between PPI status at baseline and visit was added to the model to test differences in PPI use between former and current PPI users. Linear mixed-model analysis was used as it has several advantages compared to repeated-measures ANOVA. For example, linear mixed-model analysis does not require balanced data, which are rare in real life longitudinal data, and handles missing data within the analysis. Overall, linear mixed-model analysis is a more sensitive tool for analysing repeated measures and makes more realistic assumptions of the data at hand, which results in better statistical estimations [26].

Secondly, the effect of treatment on patient reported outcomes was tested in an intention to treat analysis using a mixed effect regression model with treatment allocation, visit and an interaction term between treatment allocation and visit as exposure and symptom burden, health-related quality of life and self-rated health as outcomes, respectively, using identity as a random effect. In a follow-up analysis, PPI status at baseline and and interaction between PPI status at baseline and visit were added to the models to test differences in PROMs between former and current PPI users.

Thirdly, change in PPI use was correlated with change in PROMs between baseline visit and follow-up visit using Spearman’s correlation due to the ordinal properties of the questionnaire data.

Power calculations based on equal size of the continuous and on-demand prescription groups at an alpha level of 0.05 and a power of 0.80 using a within group standard deviation of 1.3 based on a previous study investigating changes in RDQ following PPI treatment [27] gave a minimal detectable difference between groups of ± 0.33 RDQ points. We aimed to recruit at least 600 patients to account for an expected 20% drop out rate giving the detectable difference of 0.25 in self-rated health using a standard deviation of 0.93. In total, 615 participants were enrolled in the study and CROM data were available for 605 participants and PROMs were available for 495 participants at visit 1. Number of observations included per analysis is presented in Tables 2 and 3.

In addition to the planned analyses of the trial, we also performed two exploratory analyses to give further insight into our findings. In the first exploratory analysis, esophagitis, age and obesity were tested as effect modifiers on the association between change in PPI use and change in PROMs, as these factors may influence the effectiveness of PPI treatment. Linear regression was used for the analyses, and p-values were bootstrapped using 2000 repetitions due to the ordinal properties of the questionnaire data. The model testing esophagitis as an effect modifier included PPI use, presence of esophagitis, an interaction between esophagitis and PPI use as exposures and symptom burden, health-related quality of life and self-rated health as outcomes, respectively. Information on esophagitis from endoscopy at baseline was available for 340 participants. This analysis was repeated using age group (age=/>50 years versus age < 50 years) and presence of obesity (BMI=/>30 versus BMI < 30) as effect modifiers to evaluate if age or obesity status influenced the associations. The median age was used as the cut-off and the cut-off for obesity was chosen based on the clinical relevance for GERD [28]. Age and BMI were dichotomized in these analyses to simplify the interpretation of the interaction term in the model.In the second exploratory analysis, determinants of GERD symptom burden at baseline were analysed using Spearman’s correlation for continuous variables (age, BMI, PPI consumption, health related quality of life and self-rated health) and the Mann-Whitney rank sum test for dichotomous variables (sex, smoking, PPI status, presence of esophagitis, age group and presence of obesity).

## Results

**Figure 1.**
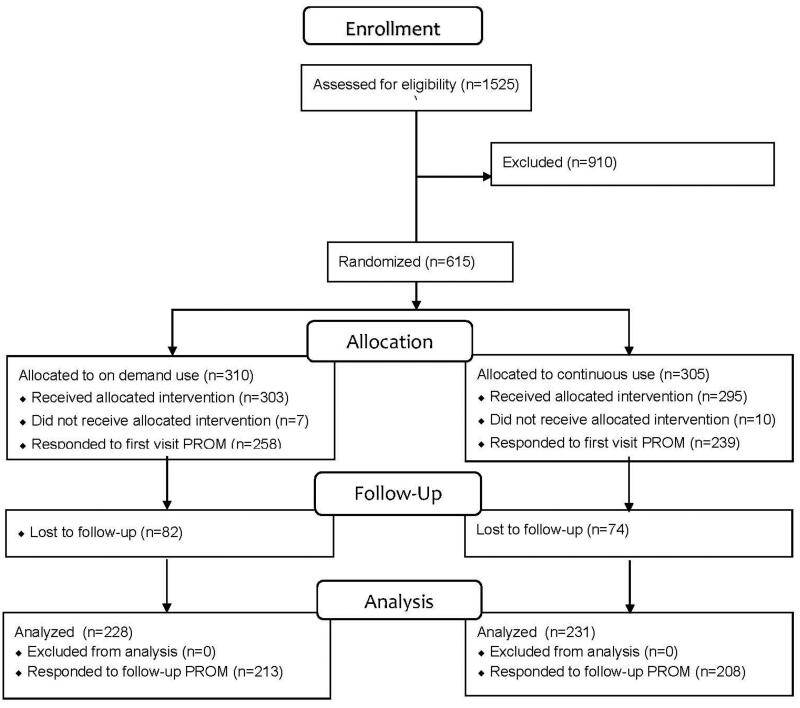
CONSORT flow chart.

A total of 615 patients, with a median age of 51 years and comprising 58% women, were enrolled in the study and randomized (Figure 1). The majority visited the primary care centre for a routine or repeat prescription review, and slightly more than half of the participants had taken PPIs during the previous week (current PPI users). The baseline CROM was completed for 605 participants, and the first PROM was filled out by 497 participants (Table 1). A total of 447 participants completed the follow-up visit, of whom 421 also filled out the follow-up PROM. There was no significant difference in study completion rates between the treatment allocations (*p* = 0.89).

**Table 1.** Baseline demographics presented by treatment allocation.

Factor	On demand	Continuous	p-value
N	310	305	
Age, median (IQR)	50 (41, 59)	51 (41, 59)	0.52
Woman	177 (57.1%)	181 (59.5%)	0.54
Current smoker	67 (22.3%)	57 (19.6%)	0.42
BMI, median (IQR)	26.6 (24.2, 29.7)	26.9 (24.1, 29.4)	0.78
Current PPI user	177 (57.8%)	179 (59.9%)	0.61
Total PPI doses last week (20 mg Omeprazole), median (IQR)	7 (0, 7)	7 (0, 7)	0.73
**Esophagitis status**			
Unknown	128 (41.8%)	136 (45.6%)	0.48
No esophagitis	127 (41.5%)	118 (39.6%)	
Los Angeles grade A	30 (9.8%)	27 (9.1%)	
Los Angeles grade B	19 (6.2%)	12 (4.0%)	
Los Angeles grade C	2 (0.7%)	5 (1.7%)	
**Country**			0.84
Greece	126 (41%)	122 (40%)	
Poland	170 (55%)	166 (54%)	
The Netherlands	8 (2.6%)	9 (3.0%)	
United Kingdom	6 (1.9%)	8 (2.6%)	
GERD symptom burden (RDQ)	1.17 (0.5, 2)	1 (0.42, 2)	0.57
Physical health (SF-12)	50.4 (41.8, 56.4)	52.1 (42.4, 56.4)	0.58
Mental health (SF-12)	45.8 (41.6, 49.9)	46.2 (42.1, 50.3)	0.82
Self-rated health	2 (2, 3)	2 (2, 3)	0.31

IQR = interquartile range (1^st^ to 4^th^ quartile), BMI = body mass index, PPI = proton pump inhibitors, GERD = gastroesophageal reflux disease, RDQ = reflux disease questionnaire, SF-12 = short form 12.

**Figure 2.**
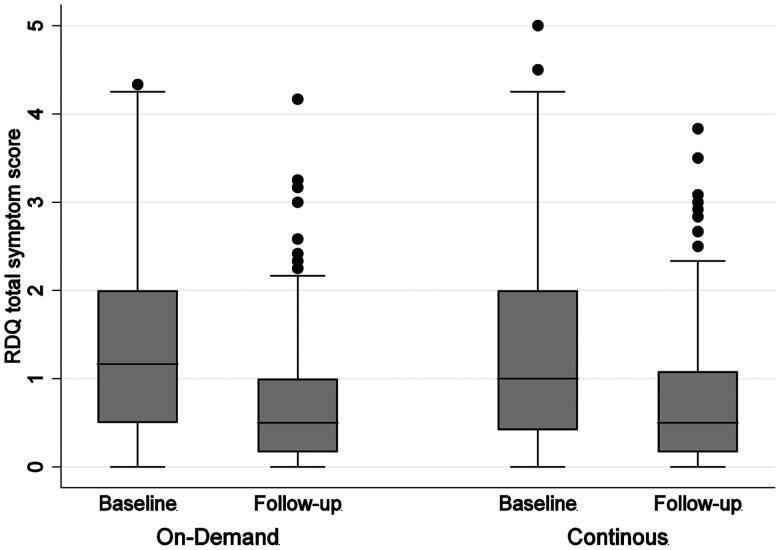
PPI use (tablets per week) at baseline and follow up between treatment allocations. PPI = proton pump inhibitors.

There was no effect of treatment allocation (i.e. continuous or on-demand prescription) on reported PPI use at follow up. However, PPI use increased between baseline and follow-up independently of treatment allocation (*b* = 1.33, *p* < 0.001, Table 2, Figure 2), which was partly due to the re-introduction of PPIs in former users. PPI consumption remained stable among current PPI users (mean weekly dose at baseline =7.7 versus follow-up *m* = 6.7) and former users increased their PPI use to a level closer to that of the current PPI users at follow-up (mean weekly dose at baseline *m* = 0 versus follow-up *m* = 5.3, *b* = 6.34, *p* < 0.001 for the interaction PPI former user at baseline and visit, Table 3). Therefore, there was no difference between treatment groups in actual drug intake, either at baseline or at follow up.

**Table 2.** Effect of treatment allocation on PPI-consumption and symptom burden, health related quality of life and self-rated health (mixed effect regression analysis).

	Observations/patients	Intercept (mean in on demand group at baseline)		Treatment allocation (difference between continuous versus on demand at baseline)		Visit (follow-up versus baseline)		Treatment allocation × Visit (Difference between continuous use group and on demand group at follow up)	
		b coefficient	95% CI	b coefficient	95% CI	b coefficient	95% CI	b coefficient	95% CI
PPI doses per week	1045/604	4.5	4.02 − 5.00	−0.06	−0.74 − 0.62	1.33***	.65 − 2.01	0.57	−0.40 − 1.53
RDQ-total score	908/497	1.30	1.19 − 1.42	−0.03	−0.19 − 0.13	−0.61***	−0.73− −0.49	0.06	−0.11 − 0.23
SF-12 Physical	506 /271	48.6	47.1 − 50.1	0.72	−1.41 − 2.85	3.31***	2.17 − 4.45	−1.54	−3.17 − 0.10
SF-12 Mental	506 /271	46.4	45.1 − 47.7	0.04	−1.83 − 1.92	0.63	−0.47 − 1.72	1.09	−0.49 − 2.67
Self-rated health	841 /423	2.19	2.07 − 2.20	0.09	−0.07 − 0.25	−0.06	−0.13-0.01	−0.05	−0.16 − 0.05

***p < 0.001.

PPI = proton pump inhibitors, RDQ = reflux disease questionnaire, SF-12 = short form 12.

**Table 3.** Effect of baseline PPI status on PPI-consumption and symptom burden, health related quality of life and self-rated health (mixed effect regression analysis adjusted for treatment allocation, visit and interaction treatment allocation and visit presented in table 2).

	Observations/participants	PPI-status (Difference between current versus former PPI user at baseline)		PPI-status × visit (Difference between current versus former PPI user at follow up)	
		b coefficient	95% CI	b coefficient	95% CI
PPI doses per week	1041/600	7.68***	7.18 − 8.18	−6.34***	−7.14−-5.61
RDQ-total score	899/492	−0.12	−0.28 − 0.05	0.22*	0.04 − 0.40
SF-12 Physical	498/267	−0.08	−2.32 − 2.15	−0.54	−2.26 − 1.18
SF-12 Mental	498/267	−0.02	−1.99 − 1.94	−0.55	−2.20 − 1.09
Self-rated health	833/419	0.21*	0.04 − 0.37	−0.08	−0.19 − 0.02

*p < 0.05.

***p < 0.001.

**Figure 3.**
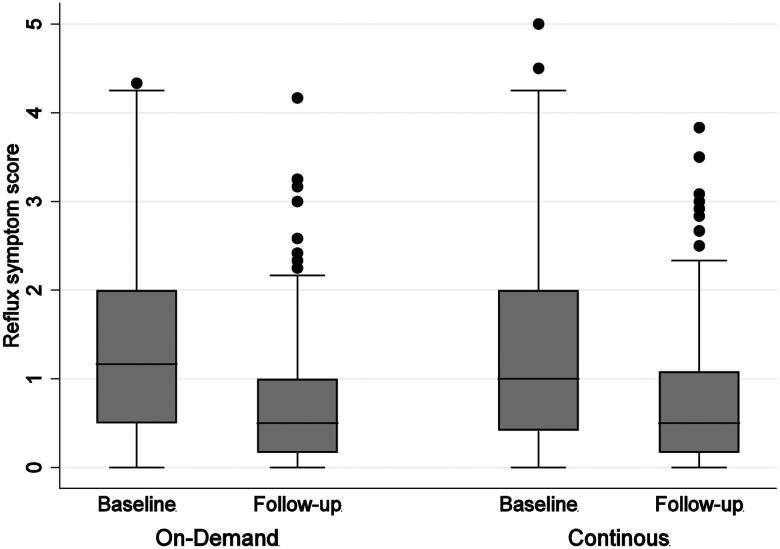
Reflux symptom burden at baseline and follow-up between treatment allocations.

There was no effect of treatment allocation on either of the patient reported outcomes at the follow-up visit (Table 2). Symptom burden and quality of life improved significantly between baseline and follow up, independent of allocation (Cohen’s D symptom burden = 0.63, Figure 3 and Cohen’s D physical quality of life = 0.37). This result was not explained by PPI status at baseline, although current PPI users, who may have had more severe disease, experienced a somewhat smaller reduction in reflux symptom burden than former PPI-users (*b* = 0.22, *p* < 0.017 for interaction PPI status at baseline and visit, Table 3).

The increased PPI intake between the baseline and follow-up visits in both treatment allocations correlated significantly with a reduced reflux symptom score (*n* = 347, ρ= −0.12, *p* = 0.02) and an improved physical health score (*n* = 217, ρ = 0.16, *p* = 0.02). However, no significant associations were found for mental health score (*n* = 217, ρ=-0.04, *p* = 0.54) or self-rated health (*n* = 354, ρ=-0.08, *p* = 0.14). Age, obesity or presence of esophagitis did not moderate the association between change in PPI intake and PROMs (data not shown).

At baseline, presence of esophagitis (*p* < 0.001), lower age (*n* = 493, ρ=-0.10, *p* = 0.02), poorer physical (*n* = 244, ρ=-0.52, p= <0.001) and mental (*n* = 244, ρ=-0.16, *p* = 0.01) quality of life, and poorer self-rated health (*n* = 417, ρ=-0.24, p= <0.001) were significantly associated with a higher reflux symptom burden. No association was found between reflux symptom burden and PPI-intake (*n* = 485, ρ=-0.04, *p* = 0.33), PPI-status (current versus former, *p*=.20), BMI (*n* = 489, ρ = 0.01, *p* = 0.72), sex (*p* = 0.65), or smoking status (*p* = 0.60).

## Discussion

This study is the first to compare the effects of on-demand versus continuous PPI prescription on symptom burden, health-related quality of life, and self-rated health in primary care patients with GERD. Participation in the study itself enhanced these outcomes, yet no differences were observed in PPI consumption or patient-reported outcomes between the on-demand or continuous PPI prescription groups at follow-up. Should treatment effects prove identical, adopting on-demand PPI therapy could reduce both treatment costs and the potential for long-term adverse effects [12]. However, although no effect of the mode of treatment was found, increased use of PPI was significantly associated with a reduced burden of reflux symptoms and improved physical health-related quality of life.

A systematic review has posited that on-demand PPI treatment is as effective for many patients with non-erosive reflux disease or mild erosive esophagitis as continuous use [11]. We had a similar population in the present study where one third of the participants had erosive esophagitis (mainly mild Los Angeles class A and B) and the rest had non-erosive reflux disease and we did not find any differences in symptom burden, quality of life, or self-rated health between treatment allocations. However, we also found no differences in PPI use between treatment allocations, which may be is due to the combination of two factors: patients who were already taking PPIs at enrolment may have been reluctant to reduce their dose when randomized for on-demand use, and the group of former PPI users who were randomized for on-demand PPI use showed an increase in use to levels similar to those of the current PPI users.

In addition, current PPI users experienced a marginally smaller reduction in reflux symptom burden compared to former PPI users, potentially due to more severe disease or a ceiling effect, where PPI usage could not be further increased in this group. The average PPI intake was not compared between the on-demand and the continuous groups in the review by Khan and colleagues, and the lack of differences in symptom burden could be attributed to the similar PPI intake between the groups.

Our findings suggest that higher PPI intake correlates with improved symptom management, regardless of treatment allocation. A recent Cochrane review [29] suggested that on-demand de-prescribing—reducing the PPI dosage—might lead to a lesser pill burden but also an uptick in GERD symptoms and a drop in patient satisfaction, underlining the importance of adequate PPI usage for maintaining symptom control in these patients.

Exploratory analyses did not support the notion that improvements in PROMs in response to increased PPI use would be moderated by age, obesity, or the presence of esophagitis. This indicates the benefits of raised PPI usage on symptom burden and quality of life are not confined to specific patient groups. Therefore, reminding GERD patients who respond well to PPIs to adhere to their medication could enhance their quality of life, irrespective of whether the prescription is on-demand or continuous, and regardless of age or the presence of esophagitis or obesity.

The improvements in symptom burden and physical health-related quality of life may be attributed to regression to the mean, as participants might have sought health care at the peak of their symptoms. However, the improvement in symptoms correlated with a specific increase in PPI use rather than a general improvement and possibly completing the symptom questionnaire may have reminded patients of their symptom burden and increased their PPI intake during the follow-up period.

This study did not gather information on hiatal hernias, *H. pylori* status or CYP2C19 genotype that may influence the effect of PPI treatment. However, the block randomization based on age and sex should limit any bias from these factors. Other limitations include missing data on symptoms and health-related quality of life of the participants who utilized the TRANSFoRm tool for questionnaires, primarily due to a technical issue, hence data loss is presumed random. Nevertheless, 421 individuals completed the follow-up PROM, which only reduced the power to detect a difference on a five-grade Likert scale from 0.25 to 0.24 and the absence of significant effect on prescription is probably not due to a lack of power. The study’s strengths lie in its sample size, making it among the few capable of discerning treatment effects of PPIs on quality of life and self-rated health. Further, it is the first pharma-independent randomized controlled trial exploring PPI treatment regimes. Future research could investigate the impacts of new acid inhibitory drugs, including Potassium-Competitive Acid Blockers (P-CAB), that may change the management of GERD in primary care.

## Conclusion

In conclusion, this real-life study found no differences between PPI users prescribed PPI on demand versus PPI users prescribed PPI continuously in terms of symptom burden, health-related quality of life, and self-rated health. Instead, we found that users prescribed PPI on demand increased their use from baseline as much as those prescribed PPI continuously, with the result that there were no differences in PPI usage between treatment allocations. Overall, symptom control improved with increased PPI use. The results are applicable in clinical practice, where health care practitioners may encourage patients with bothersome symptoms who use PPI on demand to increase their dose in order to obtain adequate symptom relief.

## Data Availability

The datasets generated during and/or analyzed during the current study cannot be made publicly available due to ethical concerns but are available from the corresponding author on reasonable request.

## References

[1] Klauser AG, Schindlbeck NE, Müller-Lissner SA. Symptoms in gastro-oesophageal reflux disease. Lancet. 1990;335(8683):1–10. doi:10.1016/0140-6736(90)90287-f. 1967675

[2] Vakil N, van Zanten SV, Kahrilas P, et al. The Montreal definition and classification of gastroesophageal reflux disease: a global evidence-based consensus. Am J Gastroenterol. 2006;101(8):1900–1920; quiz 1943. doi:10.1111/j.1572-0241.2006.00630.x. 16928254

[3] Yamasaki T, O’Neil J, Fass R. Update on functional heartburn. Gastroenterol Hepatol (N Y). 2017;13(12):725–734. 29339948 PMC5763558

[4] Ronkainen J, Aro P, Storskrubb T, et al. High prevalence of gastroesophageal reflux symptoms and esophagitis with or without symptoms in the general adult Swedish population: a kalixanda study report. Scand J Gastroenterol. 2005;40(3):275–285. doi:10.1080/00365520510011579. 15932168

[5] Gindea C, Birla R, Hoara P, et al. Barrett esophagus: history, definition and etiopathogeny. J Med Life. 2014;7(3):23–30. PMC4391409 25870690

[6] Zagari RM, Law GR, Fuccio L, et al. Dyspeptic symptoms and endoscopic findings in the community: the Loiano-Monghidoro study. Am J Gastroenterol. 2010;105(3):565–571. doi:10.1038/ajg.2009.706. 20010920

[7] Lagergren J. Adenocarcinoma of oesophagus: what exactly is the size of the problem and who is at risk? Gut. 2005;54 (Suppl 1):i1–5. doi:10.1136/gut.2004.041517. 15711002 PMC1867797

[8] Dent J, El-Serag HB, Wallander MA, et al. Epidemiology of gastro-oesophageal reflux disease: a systematic review. Gut. 2005;54(5):710–717. doi:10.1136/gut.2004.051821. 15831922 PMC1774487

[9] Andreasson A, Talley NJ, Walker MM, et al. An increasing incidence of upper gastrointestinal disorders over 23 years: a prospective population-based study in Sweden. Am J Gastroenterol. 2021;116(1):210–213. doi:10.14309/ajg.0000000000000972. 33027078

[10] Agreus L, Hellstrom PM, Talley NJ, et al. Towards a healthy stomach? Helicobacter pylori prevalence has dramatically decreased over 23 years in adults in a Swedish community. United European Gastroenterol J. 2016;4(5):686–696. 10.1177/2050640615623369 PMC5042307 27733911

[11] Khan Z, Alastal Y, Khan MA, et al. On-demand therapy with proton pump inhibitors for maintenance treatment of nonerosive reflux disease or mild erosive esophagitis: a systematic review and Meta-Analysis. Gastroenterol Res Pract. 2018;2018:6417526–6417510. doi:10.1155/2018/6417526. 30158966 PMC6109549

[12] Yu LY, Sun LN, Zhang XH, et al. A review of the novel application and potential adverse effects of proton pump inhibitors. Adv Ther. 2017;34(5):1070–1086. doi:10.1007/s12325-017-0532-9. 28429247 PMC5427147

[13] Sandhu DS, Fass R. Current trends in the management of gastroesophageal reflux disease. Gut Liver. 2018;12(1):7–16. doi:10.5009/gnl16615. 28427116 PMC5753679

[14] DeSalvo KB, Fan VS, McDonell MB, et al. Predicting mortality and healthcare utilization with a single question. Health Serv Res. 2005;40(4):1234–1246. doi:10.1111/j.1475-6773.2005.00404.x. 16033502 PMC1361190

[15] DeSalvo KB, Bloser N, Reynolds K, et al. Mortality prediction with a single general self-rated health question. A meta-analysis. J Gen Intern Med. 2006;21(3):267–275. doi:10.1111/j.1525-1497.2005.00291.x. 16336622 PMC1828094

[16] Idler EL, Benyamini Y. Self-rated health and mortality: a review of twenty-seven community studies. J Health Soc Behav. 1997;38(1):21–37. 9097506

[17] Mastellos N, Blizniuk G, Czopnik D, et al. Feasibility and acceptability of TRANSFoRm to improve clinical trial recruitment in primary care. Fam Pract. 2016;33(2):186–191. 26711958 10.1093/fampra/cmv102

[18] Ethier JF, Curcin V, McGilchrist MM, et al. eSource for clinical trials: implementation and evaluation of a standards-based approach in a real world trial. Int J Med Inform. 2017;106:17–24. 28870379 10.1016/j.ijmedinf.2017.06.006

[19] Lim SN, Keung C, Zhao L, et al. TRANSFoRm: implementing a learning healthcare system in Europe through embedding clinical research into clinical practice. *48th Hawaii International Conference on System Sciences IEEE*; 2015.

[20] Saganowski S, Misiaszek A, Brodka P, et al. TRANSFoRm eHealth solution for quality of life monitoring. AMIA Jt Summits Transl Sci Proc. 2016;2016:231–239. 27570677 PMC5001750

[21] Jankowski J, Saganowski S, Bródka P. Evaluation of TRANSFoRm mobile eHealth solution for remote patient monitoring during clinical trials. Mobile Inform Syst. 2016;2016:1–16. doi:10.1155/2016/1029368.

[22] Mastellos N, Andreasson A, Huckvale K, et al. A cluster randomised controlled trial evaluating the effectiveness of eHealth-supported patient recruitment in primary care research: the TRANSFoRm study protocol. Implement Sci. 2015;10(15). 10.1186/s13012-015-0207-3 PMC4318251 25648301

[23] Shaw MJ, Talley NJ, Beebe TJ, et al. Initial validation of a diagnostic questionnaire for gastroesophageal reflux disease. Am J Gastroenterol. 2001;96(1):52–57. 11197287 10.1111/j.1572-0241.2001.03451.x

[24] Brazier JE, Harper R, Jones NM, et al. Validating the SF-36 health survey questionnaire: new outcome measure for primary care. BMJ. 1992;305(6846):160–164. doi:10.1136/bmj.305.6846.160. 1285753 PMC1883187

[25] Ganna A, Ingelsson E. 5 Year mortality predictors in 498,103 UK biobank participants: a prospective population-based study. Lancet. 2015;386(9993):533–540. doi:10.1016/S0140-6736(15)60175-1. 26049253

[26] Shek DT, Ma CM. Longitudinal data analyses using linear mixed models in SPSS: concepts, procedures and illustrations. Sci World J. 2011;11:42–76. doi:10.1100/tsw.2011.2. PMC5719989 21218263

[27] Shaw M, Dent J, Beebe T, et al. The reflux disease questionnaire: a measure for assessment of treatment response in clinical trials. Health Qual Life Outcomes. 2008;6(1):31. doi:10.1186/1477-7525-6-31. 18447946 PMC2390523

[28] El-Serag HB, Ergun GA, Pandolfino J, et al. Obesity increases oesophageal acid exposure. Gut. 2007;56(6):749–755. doi:10.1136/gut.2006.100263. 17127706 PMC1954847

[29] Boghossian TA, Rashid FJ, Thompson W, et al. Deprescribing versus continuation of chronic proton pump inhibitor use in adults. Cochrane Database Syst Rev. 2017;3: CD011969. 28301676 10.1002/14651858.CD011969.pub2 PMC6464703

